# Implementing a ‘Vegetables First’ Approach to Complementary Feeding

**DOI:** 10.1007/s13668-022-00399-z

**Published:** 2022-02-12

**Authors:** Chandani Nekitsing, Marion M. Hetherington

**Affiliations:** 1 Department of Health Sciences, University of York, York, YO10 5DD UK; 2 School of Psychology, University of Leeds, Leeds, LS2 9JT UK

**Keywords:** Vegetables, Complementary feeding, First foods, Food preference, Early exposure, Healthy eating, Eating behaviour, Weaning, Infant feeding, Child eating

## Abstract

***Purpose of Review*:**

To provide a rationale for promoting a vegetables first approach to complementary feeding (CF), building on prior exposure to vegetable flavours experienced in utero and via breastfeeding (chemosensory continuity).

***Recent Findings*:**

Vegetables confer selective health benefits but population intakes are below recommendations globally; maternal intake of vegetables during both pregnancy and lactation promotes familiarity with some vegetable flavours. Building on this exposure, vegetables as a first food during CF further promote acceptance. However, experiments testing efficacy of a vegetables first approach to CF demonstrate increased liking and intake, some evidence of generalisability but little evidence of sustained effects beyond infancy.

***Summary*:**

The aim to increase the quantity and variety of vegetables eaten by children is both desirable, to improve nutrient quality of the diet, and achievable. However, longer, larger, randomised control trials are needed to evidence any longer term, sustainable benefits to liking and intake of vegetables.

## Introduction

In common with other mammals, the sole source of nutrition for the human neonate after birth is milk. Human milk provides essential nutrients required for early growth and development as well as a sophisticated endocrine signalling system, bioactive agents promoting immunity and complex flavours derived from the maternal diet [1]. The World Health Organization (WHO) recommends exclusive breastfeeding to 6 months and to extend this for another 2 years (and more) as complementary foods are offered to infants. Whilst there is clear guidance about when to introduce solid foods and how to recognise signs of readiness in the infants from government agencies and the WHO [2], the recommendations for which foods to offer vary by country [3]. There is little consensus on first foods to offer infants [3], since this will depend on local food customs, availability, accessibility and affordability of these foods. Which foods to offer might also depend on which flavours are more acceptable to infants at the time of complementary feeding. Rigorous investigation of infants’ response to the five basic tastes at 3 m, 6 m and 12 m reveals that sweet and salty tastes were most accepted whereas sour and bitter tastes were least accepted, but not systematically rejected [4]. Therefore, at the time of complementary feeding, offering vegetables, which may have a bitter flavour, can be a safe source of nutrients. Furthermore, intake of vegetables has selective health benefits if sustained in the long term. Thus, in this review, we make the case for a vegetables first approach to complementary feeding (CF).

The argument for this approach is made by addressing the following three research questions: (1) why is vegetable intake important; (2) how is exposure to vegetable flavours achieved before complementary feeding; (3) does early exposure to vegetables produce long-term preferences and changes in the diet? The review then concludes with evidence-based strategies for achieving the goal of increasing vegetable consumption using a vegetables first approach to CF and proposed future studies.

## The Omnivore’s Paradox

**Fig. 1:**
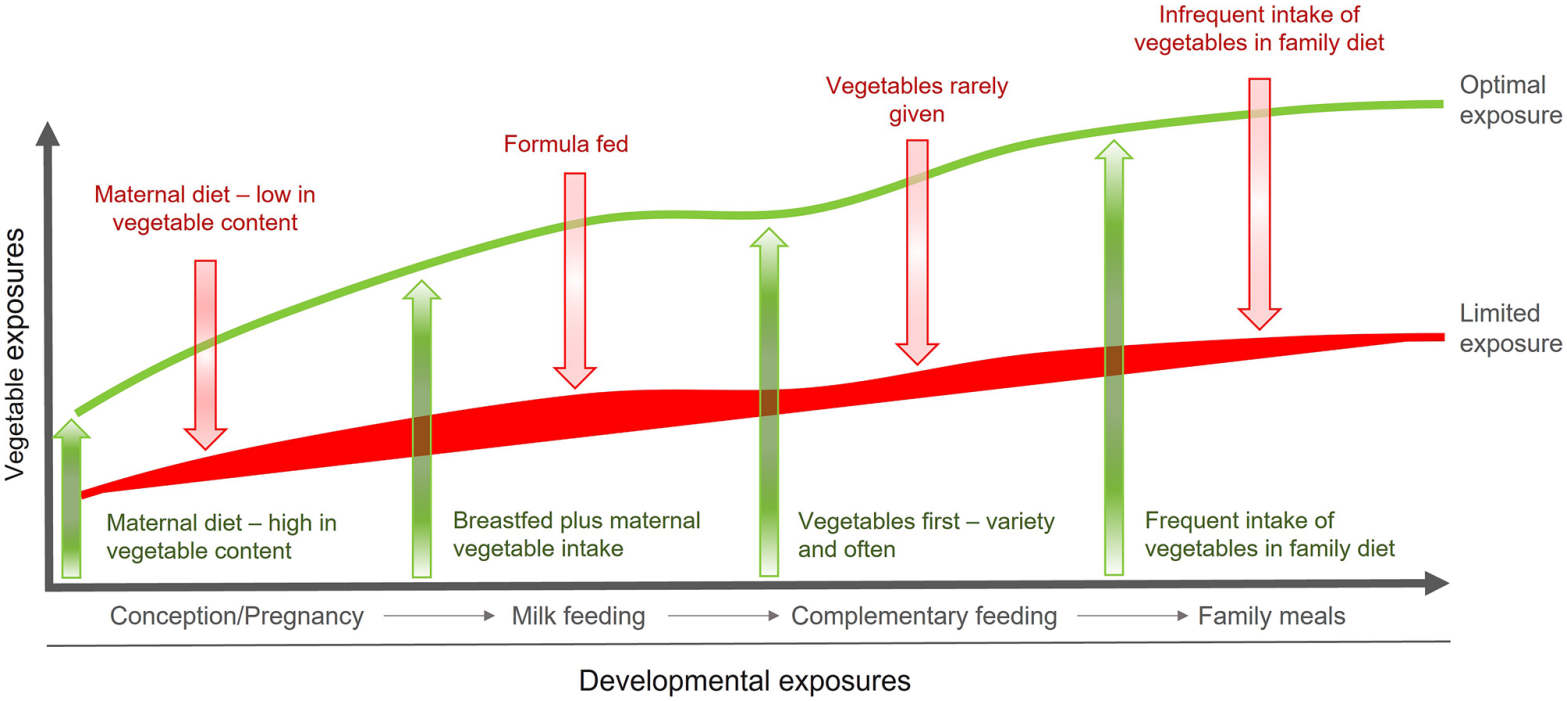
Illustration of chemosensory exposure from conception to the family diet, with the ideal number of exposures represented in green ink and the less optimal number of exposures in red ink. Clearly, some infants will have exposures which vary according to maternal behaviours in early life (pregnancy, lactation, CF) and later exposures from the family diet/external environment

During complementary feeding, infants transition from their status as univore to omnivore, in what has been termed by Paul Rozin [5], the ‘omnivore’s paradox’. The infant requires a varied diet for optimal health and growth, but consuming novel foods presents a potential hazard. Therein lies the paradox, the transition to an omnivorous diet involves overcoming a natural reluctance to accept new substances which may pose a threat to the infant. This transition is facilitated by exposure in utero to flavours derived from the maternal diet and then through the flavours of breastmilk termed ‘chemosensory continuity’ [6]. By the time solid foods are offered at around 6 months of age during complementary feeding (CF), the infant is already familiar with specific odour and flavour components of the maternal diet which they favour over other odours [7]. The concept of chemosensory continuity can be described as link between the mother’s food choices during the first 1000 days of life, expanding out towards the family diet (see Fig. 1).

Evidence reviewed by Harris and Mason [8] identifies 4 to 6 months of age as a ‘sensitive period’, in which to offer novel flavours. Whilst it is essential that infants make this transition to solids since breastmilk alone may be insufficient to meet the energy demands of the baby beyond 6 months [9], the potential risks associated with this may be substantial, in some contexts. For example, there is an increased risk of malnutrition related to poor nutritional quality of foods offered and a greater chance of microbial contamination from solid foods leading to infection. Malnutrition and infection-related inadequate intake and illness are significant contributors to high infant mortality rates in the under fives [10]. Therefore, when infants are ready to accept solid foods, in large parts of the world, introducing complementary foods is challenging as a result of food insecurity and risky due to lack of access to clean water for hygienic food handling. For all caregivers, the transition to solids is an important milestone and for many, in low resource parts of the world, the source of considerable anxiety.

**Fig. 2:**
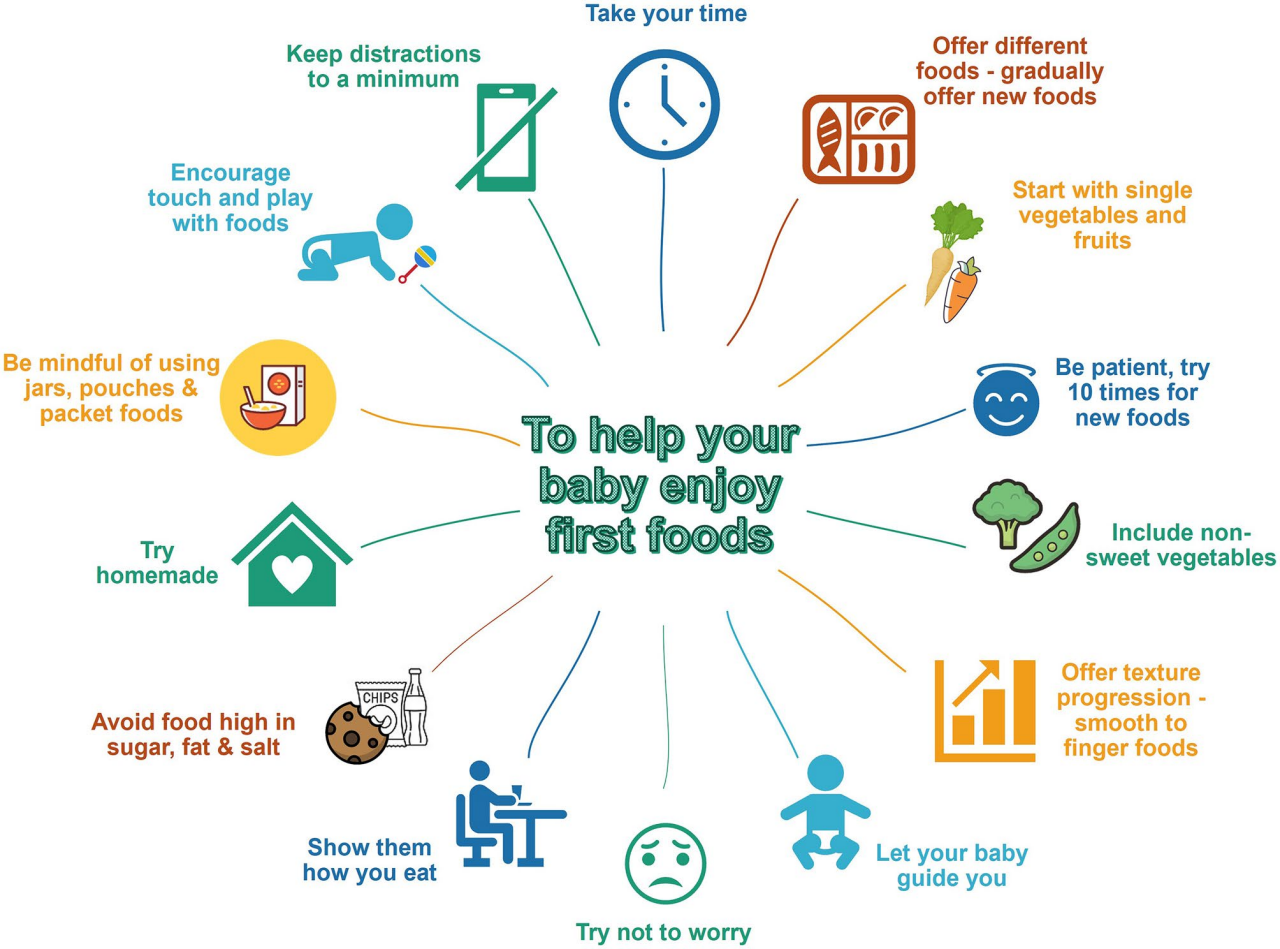
Graphical summary of the National Health Service (NHS) UK guidance on introducing first foods to babies [14]

Guidance suggests that solid foods are introduced when infants show physical signs of readiness. National guidelines for CF encourage caregivers to look for developmental signs such as holding the head up, reaching for food and loss of the tongue extrusion reflex (e.g. WHO [11]). In the USA, the American Academy of Nutrition and Dietetics advises that first foods should include ‘puréed meats, poultry, beans and iron-fortified cereals’, with encouragement to offer ‘one new single-ingredient food at a time’ [12]. The Dietary Guidelines for Americans (DGS) 2020–2025 [13] also advocate iron-rich first foods and encourage a variety of foods drawn from the four main food groups (protein foods; vegetables and fruits; dairy and grains) with advice that infants may need multiple exposures to develop acceptance. They discourage added sugar, foods high in sodium, honey and unpasteurised foods and beverages. In the UK, the National Health Service (NHS) suggests offering first foods around 6 months of age and to start with single vegetables and fruits, introducing texture progression from purées to mashed, lumpy and finger foods. The NHS guidance encourages dietary diversity achieved by offering a variety of flavours to try, progressing to new foods gradually and being patient with multiple exposures to new foods to establish acceptance (see Fig. 2 for a graphical summary).

It is known that early exposure is emerging as a promising means to encourage liking and acceptance of vegetables [15]. There appears to be a sensitive period to present vegetables as a first food, since this is an important component of a balanced diet, yet children across the world are eating too few vegetables. In the next section, the rationale for promoting vegetable intake is considered within the context of selective health benefits.

## Why Are Vegetables Important?

A global shift towards a plant-based diet is necessary to counteract climate change according to the United Nations, since agrarian practices are less polluting and less water intensive than raising livestock [16]. The EAT-Lancet commission’s report on planetary health emphasises a diet with a greater proportion of whole grains, fruits, vegetables, nuts and legumes than animal and dairy components. Thus, two major global pressures are coalescing to encourage sustainable plant-based diets for human health and environmental sustainability [17••].

To promote dietary health, the World Health Organization recommends intake of at least five servings (400 g) of fruits and vegetables (F&V) per day [18]. However, in Sub-Saharan Africa, intakes fail to reach 200 g per day [19] and it is estimated that 27% of deaths are attributed to low consumption of F&V [20]. Globally, only half (52%) of young children meet the minimum meal frequency and less than one-third (29%) achieve the minimum dietary diversity threshold, with large disparities across and within regions [21]. Therefore, low meal frequency increases risk of malnutrition and lack of dietary diversity increases risk of micronutrient deficiency.

Vegetables contribute to a balanced diet for children providing nutrients essential to development and health [22, 23]. UNICEF [24] reports that more than half of the poorest children aged 6 to 23 months are not offered fruits or vegetables. In general, when children are offered these foods, they tend to eat more fruits than vegetables [25•, 26–28]. In comparison to vegetables, fruits tend to be well-liked due their sweet taste, and are generally more readily available and accessible (can be eaten with limited preparation). Intakes of dark green vegetables are especially low among infants according to the Feeding Infants and Toddlers Survey (FITS) from the USA [29].

Micronutrient deficiency (MD), or ‘hidden hunger’, is critically important in children since they require sufficient energy and micronutrients to achieve optimal growth and health, and in the longer term, MD increases the risk of chronic diseases such as heart disease and cancer [30]. Specific health benefits of consuming fruits and vegetables are attributable to bioactive ingredients such as vitamin (e.g. vitamins A, C, E, K, folic acid), mineral (e.g. magnesium, calcium, zinc, potassium) and fibre content as well as, most importantly, phytochemicals (e.g. flavonoids, carotenoids) producing anticarcinogenic and anti-inflammatory effects [31]. Epidemiological studies indicate that diets rich in green leafy, β-carotene and vitamin C containing F&V reduce coronary heart disease risk [30], with a specific health benefit linked to eating vegetables [32]. Dietary phytochemicals suppress oxidative stress-induced DNA damage, which is a major contributor to pathogenesis and neurodegenerative disease progression. In humans, approximately one serving of green, leafy vegetables eaten each day was associated with a slower rate of cognitive decline in a prospective study of ageing [33]. Intake of specific vegetables with their potent antioxidant action produces functional benefits to physical and cognitive health, and is particularly important for children living in the global south.

Therefore, eating a well-balanced diet, rich in F&V, is known to reduce risk of developing various non-communicable diseases [23, 34–36]. To achieve health promoting benefits, sufficient amounts of plant-based foods must be eaten and consumed in a wide variety. Vegetables are considered more beneficial than fruits because they are higher in fibre content and lower in sugar, sodium, fat, cholesterol and energy density [31, 37].

Despite the known benefits of eating a range of vegetables, challenges persist in increasing vegetable intake compared to fruit intake [38]. There are many reasons for low consumption of vegetables in children, relative to fruits, ranging from lack of access and provision, higher costs of vegetables and a universal dislike of vegetables linked to their sensory properties, e.g. bitter flavour and/or unfamiliar texture [39]. Hence, early exposure to vegetables, during complementary feeding, is an ideal time to encourage children to accept and to like vegetables, before eating habits are more established and harder to change [40].

In summary, vegetable intakes remain low despite their selective health and environmental benefits as part of a plant-based diet. Therefore, promoting vegetable intakes during the first 1000 days from conception could be a means to ensure familiarisation even before solid foods are introduced.

## Flavour Exposure Before Complementary Feeding

Foetal exposure to flavours derived from the maternal diet is achieved through amniotic fluid. Flavours are composed of complex substances including volatile compounds. It is these volatiles that contribute to the distinctive flavour fingerprint that permits animals and humans to recognise a suitable food source and to distinguish them from non-edible or harmful substances. Whilst flavours are distinctive, the volatiles which contribute to flavour are shared across foods, for example garlic, onions and mustards, as well as some other vegetables, share the volatiles allyl-isothiocyanate and allicin [41]. Therefore, some flavours derived from the maternal diet are transferred to the developing foetus through swallowing amniotic fluid. Experimental evidence across species indicates that foetal exposure facilitates recognition and response to familiar odours and flavours. One such study was conducted by Hepper [42] who exposed pregnant rats to garlic or no garlic, then presented garlic or onion in Petri dishes to 12-day old pups. Rat pups from mothers who had been fed garlic preferred garlic over onion and this finding was replicated in cross-fostered pups. Foetal learning appears to be a biologically adaptive behaviour and provides an important connection between the mother and her offspring. This connection is made via chemosensory continuity between the maternal diet, foetal learning and lactation which is then expressed through preferences of offspring, serving kin recognition [43] and signalling safe sources of edible substances.

Marlier and Schaal [7] demonstrated that human neonates are capable of detecting, discriminating and orienting towards odours derived from human milk compared to formula. The authors conducted a series of experiments and found that babies who were breastfed (experiment 1) and formula fed (experiments 2 and 4) demonstrated more persistent head-orientation and mouthing responses to human milk odour compared to formula milk odour. For example, in the second experiment, 12 exclusively formula-fed neonates were exposed to odours of unfamiliar human milk and unfamiliar formula milk (not the brand they had ingested since birth). Babies oriented longer to the odour of unfamiliar human milk compared to an unfamiliar formula milk. These findings demonstrate that human milk is attractive to the newborn, even if they have been formula fed, most likely since human milk contains aromatic compounds familiar to infants via transmission in utero [7]. This is a profound observation since it suggests that the attractiveness of human milk is greater than the experiential learning produced by formula feeding. It also illustrates that the complexity of human milk provides an advantage to the breastfed baby over formula in promoting acceptance of novel foods.

A substantial body of research by Mennella and her colleagues provides evidence of chemosensory continuity from the maternal diet to amniotic fluid and lactation [44–49]. In a randomised control trial, Mennella and her colleagues assigned mothers to consume vegetable juices (beetroot, celery and carrot) once per day for 1 month during breastfeeding beginning at 0.5, 1.5 or 2.5 months postpartum, or for a longer duration of 3 months beginning at 0.5 months [50••]. The earliest exposure postpartum (0.5 months) for 1 month duration produced a significant shift in intake of cereal flavoured with carrot and produced a faster rate of eating this cereal than infants who were exposed later or for longer. This elegant study confirms chemosensory learning and suggests that the early timing is more important than duration of exposure during lactation.

Only some flavours have been explored in the context of research and evidence for experience of vegetable flavour in the amniotic fluid or breastmilk and subsequent vegetable intake is limited. To date, only a few vegetable flavours have been tested, namely carrot, garlic and mixed vegetable juice (carrot, beet, celery). It is not known whether experience of one vegetable flavour will generalise to intake to other vegetables. For example, a randomised control trial (RCT) conducted by Mennella et al. [50••] found that the timing of when the mixed vegetable juice (carrot, celery, beet) was introduced to the mother affected their child’s acceptance of carrot intake. Thus, introduction in the earlier months (around 1 month) during lactation had a greater effect than exposure around 3 months. However, the study found no generalisation effect of the vegetable juice on infant’s intake of an unexposed flavour (broccoli). Further study is warranted to investigate transferable and sustainable effects of maternal vegetable intake during breastfeeding on later preferences for vegetable acceptance and preference in children [51]. Mothers report changes in appetite and food aversions, including eating fewer vegetables during pregnancy [52]; therefore, advice to eat more vegetables must be given without undue pressure.

Evidence suggests that there are long-term dietary and health benefits of mothers eating vegetables during pregnancy and lactation. In addition to the direct health benefits to the mother, early exposure to vegetable flavours is likely to establish familiarity and increase willingness to accept these flavours during complementary feeding. However, for children to learn to like and accept a range of vegetables, they need more direct exposure, repeated over time.

## Vegetables as a First Food – Effects on Liking and Intake

Early exposure to vegetables flavours during foetal development and then lactation promotes familiarity via chemosensory continuity. Progressing this to solid food introduction is the obvious next step. However, unlike other foods, vegetables are disliked due to their bitter taste and sometimes unfamiliar texture, this has been described as a ‘hard-wired’ dislike [53].

Direct experience of food flavour begins during complementary feeding [54]. Infants are more willing to accept different food flavours and textures during CF than later on. There appears to be a critical period for introducing different textures to infants before 9 to 10 months. Data from the ALSPAC study shows that delaying texture progression beyond this time is linked to feeding difficulties including children consuming a limited variety of foods [55, 56]. In addition, around 24 months children’s neophobia and food fussiness appear [57]. Food neophobia is the fear of trying new foods and is associated with food fussiness, which is defined as follows: highly selective eating, increased food rejection (including familiar/previously liked foods) and being less willing to taste unfamiliar foods [58]. These traits are observed minimally in infancy but tend to peak between 2 and 6 years of age [58–60] and have been associated with lower intakes of vegetables [61, 62]. However, with repeated exposure (taste/visual), fussiness and neophobia may be moderated [58, 63, 64].

There have been a small number of trials testing the impact of a ‘vegetables first’ approach to complementary feeding (CF) and at least two current, larger scale trials have been registered in the Netherlands [65] and New Zealand [66] to test this approach.

A vegetables first approach to increase intake of vegetables was investigated by Gerrish and Mennella [67]. In this study, formula-fed infants who had earlier been introduced to cereal at the beginning of CF were randomised at age 4.5 to 5 months to receive puréed carrot, potato or a variety of vegetables (peas, potatoes and squash) every day for 9 days. On days 1 and 11, all babies received carrot and on day 12, they received puréed chicken. On these days, foods were given in the laboratory where intake was filmed and weighed. Infants who had been fed carrots or the variety of vegetables consumed more carrot on day 11 than the infants who were fed potato. The variety group also consumed more of the novel food (chicken) than the other groups and prior daily fruit exposure increased the initial intake of carrot on day 1. This study demonstrated three important findings: firstly, that offering carrot or a variety of vegetables (including carrot) early on in CF enhanced acceptance of carrot; secondly, that vegetable variety increased willingness to consume a novel food and thirdly, that sweet taste experience via fruit intake elsewhere in the diet increased intake of a sweet vegetable – carrot, during the first exposure to this food.

A number of studies have confirmed that this approach increased vegetable acceptance in the short term [67–69]. Barends et al. [70] tested a vegetables first approach within a RCT in the Netherlands, where mothers were assigned to offer either puréed vegetables or fruits as first foods every other day for 18 days at the beginning of complementary feeding. For one vegetable group, the target vegetable was green bean and for the other, it was artichoke. For one fruit group, the target was apple or plum. On days 1 and 2 and days 17 to 19, foods were given to the infants in the laboratory so that responses could be filmed, and intakes weighed and recorded. On days 3 to 16, purées were given to infants at home. On day 19, infants in the vegetable groups were given their first taste of fruit purée and infants in the fruit groups were given their first taste of vegetable purée. Intake of both vegetable and fruit increased with repeated exposure but intake of fruit was always higher than vegetable from the beginning of the study. Exposure to fruit purée had no effect on intake of vegetable purée and subsequent follow-up when the infants were 12 and then 23 months of age revealed that a vegetables first approach increased daily vegetable intake at 12 months but was no longer observed at 23 months [70]. Furthermore, intake of vegetables correlated with maternal ratings of liking for vegetables. No differences were found for fruit intake. Overall, this trial demonstrated that offering vegetables first confers a benefit to infants to promote vegetable intakes early on, but this may be moderated by whatever other foods are then introduced by mothers after the trial since the preferential effects of vegetables first did not endure beyond 1 year.

Within the HabEat study across Europe, a ‘vegetables first’ approach was trialled in three different nations – Greece, Portugal and the UK. The intervention groups received guidance to introduce a variety of vegetables as first foods during the first 15 days of the complementary feeding period and the control group received the standard complementary feeding guidance from that country [71]. Mothers were provided some commercially available vegetable purées but could prepare their own if preferred. They were asked to offer the selected five vegetables in a sequence over 15 days, e.g. ABCDE, repeated three times and to record compliance. After this period, for a further 5 days, parents continued to offer vegetables and to introduce age-appropriate foods to the diet. All infants were tested 1 month after the intervention to assess acceptance (liking and intake) of a new vegetable, namely artichoke. Infants in the intervention groups ate significantly more of the unfamiliar vegetable than those in the control group, and infants from the UK consumed almost double the amount of the new vegetable compared to control group. This effect was not observed in infants from Greece or Portugal, perhaps because vegetables (and vegetable soup in Portugal) are already a common first food [71]. In a country like the UK, a vegetables first approach increased liking and intake of a novel vegetable compared to usual practice, which at the time was baby rice. Whereas in a country like Portugal, most of the infants assigned to the control group (70%) had already received vegetable soup as their first food and therefore benefitted from prior vegetable exposure.

Hetherington et al. [15] tested the effect of offering vegetables first in a step-by-step approach to complementary feeding. In this RCT, mothers who were ready to introduce solid foods to their babies (mean age 5 months) were given jars of a variety of single, pure vegetables to offer their infant on a standard rotation (carrot, green bean, spinach, broccoli). The vegetables were added to breast or formula milk, each day for 12 days and then the same rotation of puréed vegetables was added to baby rice twice a day for 12 days. By day 25, infants in the intervention had a greater exposure to the pure vegetable flavour added to milk then rice, than infants in the control group who received plain milk and rice. On days 25 and 26, mothers brought their infants to the laboratory so that their intake, liking and rate of eating of pure vegetables (without milk or rice added) could be recorded (and facial responses filmed). This was then repeated at days 33 and 34. Intake, liking and rate of eating the pure vegetables were greater for the intervention than the control group, and greater for the sweet vegetable (carrot) than for green beans [15]. However, any significant differences by group did not endure at the follow-up measurements when the infants were 12 and 24 months of age. By this time, children would have had widespread exposure to vegetables as part of the family diet.

A vegetables first approach was proposed via a consensus statement organised through the British Nutrition Foundation [72]. This report considered the evidence sufficient to start recommending vegetables first during complementary feeding as a means to increase vegetable acceptance early on with potential benefits to later preferences and intake. This approach has been included within guidance from the UK through the Scientific Advisory Committee for Nutrition [73], the National Health Service [14, 74] and ESPGHAN [75].

The evidence on which these recommendations were based, whilst sufficient, did not include larger, longer RCTS to strengthen the evidence base. Indeed, most guidance continues to recommend fruit, vegetables, meat and iron-rich infant cereals as first complementary foods (e.g. WHO [2]). The aim to increase the quantity and the variety of vegetables eaten by children remains desirable to improve nutrient quality of the diet and is achievable [68]. Further research is needed to strengthen the evidence base and to investigate ‘best practice’ strategies to promote vegetable intake in early life.

## Discussion

In conclusion, vegetable intake is beneficial to long-term health but in many countries across the world, children fail to eat enough food in general and sufficient vegetables in particular. There are windows of opportunity for vegetable exposure during pregnancy to promote optimal flavour exposure in the first 1000 days from conception to 24 months. Evidence that maternal diet can influence flavour experience is promising and breastmilk transfers flavours to infants. Vegetables as a first food, where this is affordable, accessible and feasible could be incorporated into the guidance according to Bell et al. [76••]. The vegetables first approach is an easy message to follow for parents but despite the benefits of offering vegetables during CF, this is not a common practice in many countries. Policy change and government recommendations to support a vegetable first approach to CF are needed. However, adherence to this guidance and to health recommendations in general may be influenced by external factors such as affluence, access, affordability, education and culture [77••]. Hence, parental guidelines for CF should be sensitive to these factors and, where possible, tailored to suit family circumstance. Also, given the sensitive period of 6 months for introducing novel vegetables during CF, this presents an opportunity for the entire family to adopt a more healthy eating pattern [78].

However, not all food products developed for complementary feeding are equally nutritious. Caregivers often rely on readily available commercial products for first food introduction. Whilst these are convenient and safe for babies, a number of studies systematically analysing the nutrient and energy content of baby foods in jars, pouches and packets have demonstrated high sugar content even when products are described as containing vegetables [79, 80••, 81•]. The Nutrient Profile Model (NPM) developed by WHO could be adopted widely to check the nutrient quality of commercially available baby foods, especially if chosen for the vegetables first approach (https://babyfoodnpm.org/). Those baby foods which fall below the quality standards presented in the NPM (e.g. exceeding acceptable levels of total fat, total sugars or salt) have been identified as targets for marketing restrictions [82] and so food companies are encouraged to use reformulation to improve nutrient standards. There are negative consequences of consuming excess sugar in the diet from a nutritional and dental perspective, but if infants are accustomed to sweetness in foods, this may also preclude the development of liking and intake of vegetables which are generally bitter and less liked than fruits [83].

Longer, larger interventions are needed to investigate the sustainable benefits of earliest flavour experiences through maternal diet to breastmilk and to complementary feeding for increasing vegetable intake in infancy and beyond. Similarly, the generalisability of the vegetables first approach and any transferable benefits of vegetable exposure in pregnancy (e.g. the Dastatuz project [84]) to other, unfamiliar vegetables to the quality of the overall diet should be investigated.

In summary, a ‘vegetables first, frequently and in variety’ approach provides the first steps towards long-term acceptance and preference for vegetable. We propose that where feasible, vegetable intake should be encouraged in pregnancy and lactation and a vegetables first approach to complementary feeding supported by public health agencies. If encouraged worldwide, a vegetables first approach could contribute to a healthier start for all children.
